# Fundamental differences between glassy dynamics in two and three dimensions

**DOI:** 10.1038/ncomms8392

**Published:** 2015-06-12

**Authors:** Elijah Flenner, Grzegorz Szamel

**Affiliations:** 1 Department of Chemistry, Colorado State University, Fort Collins, Colorado 80523, USA

## Abstract

The two-dimensional freezing transition is very different from its three-dimensional counterpart. In contrast, the glass transition is usually assumed to have similar characteristics in two and three dimensions. Using computer simulations, here we show that glassy dynamics in supercooled two- and three-dimensional fluids are fundamentally different. Specifically, transient localization of particles on approaching the glass transition is absent in two dimensions, whereas it is very pronounced in three dimensions. Moreover, the temperature dependence of the relaxation time of orientational correlations is decoupled from that of the translational relaxation time in two dimensions but not in three dimensions. Last, the relationships between the characteristic size of dynamically heterogeneous regions and the relaxation time are very different in two and three dimensions. These results strongly suggest that the glass transition in two dimensions is different than in three dimensions.

## Abstract (web summary)

The existing glass transition theories show little dimensional dependence. Here Flenner *et al*. disprove this general consensus by finding that, for instance, the bond-orientational relaxation is decoupled from the translational relaxation in two dimensions, but not in three dimensions.

## Graphical abstract

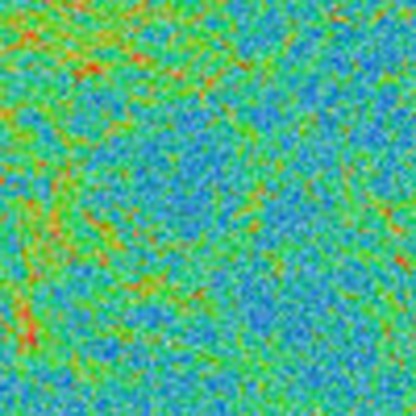

In two-dimensional (2D) solids, thermal fluctuations destroy crystalline order, displacement correlations increase logarithmically and density correlations decay according to power laws^1^,^2^. However, there can be long-range bond-orientational order in 2D^2^. The transition from the 2D fluid phase to the solid phase can occur in two steps with an intermediate phase characterized by an exponential decay of the density correlations and a power-law decay of the bond-orientational correlations^1^,^3^. In contrast, in three-dimensional (3D) solids, fluctuations do not destroy crystalline order^4^, and long-range translational and rotational order emerge together at the freezing transition.

Despite these differences between 2D- and 3D-ordered solids, the formation of an amorphous solid on supercooling a fluid, that is, the glass transition, is generally assumed to have similar characteristics in 2D and 3D^5^. This assumption is reflected in the trivial dimensional dependence of most glass transition theories^6^.

We show that structural relaxation of supercooled fluids in two dimensions is different than in three dimensions. While we find the transient localization often associated with glassy dynamics in three dimensions, we do not find any transient localization in two dimensions if we simulated systems large enough to remove any finite size effects. Furthermore, the temperature dependence of the bond-orientational correlation time is decoupled from that of the translational relaxation time in two dimensions, but these relaxation times have very similar temperature dependence in three dimensions. Along with these differences in structural relaxation, we also find that the characteristic size of regions of correlated mobility, dynamic heterogeneities, increases faster with the structural relaxation time in two dimensions than in three dimensions, and these regions are more ramified in two dimensions than three dimensions. Last, we show that the structural relaxation and heterogeneous dynamics depends on the underlying dynamics in two dimensions.

## Results

### Structural relaxation

To demonstrate the differences between glassy dynamics in two and three dimensions, we focus on two closely related glass-forming fluids: the 3D 80:20 binary Lennard–Jones system introduced by Kob and Andersen^7^, and its 2D variant that has the same interaction potentials but a 65:35 composition to avoid crystallization^8^. To simulate the relaxation in these systems, we used the standard Newtonian dynamics^9^. We also simulated the 2D system using Brownian dynamics^9^ and we comment on the differences between these two dynamics. See Methods for the simulation details and the reduced units used to present the results. We examined three other 2D glass formers and one additional 3D glass former (see Methods for details of the systems). We present some results for the additional 2D glass formers in the main text and in Supplementary Material. The figures showing results for the three additional glass formers are labelled with the labels given in Methods, and figures without labels show results for the Kob–Andersen mixtures.

**Figure 1:**
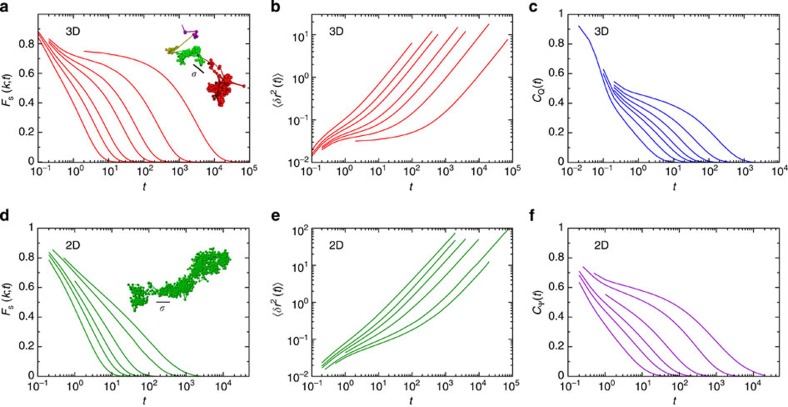
Structural relaxation in two and three dimensions. (**a**) The self-intermediate scattering function *F*_s_(*k*; *t*) for the 3D glass former for *T*=1.0, 0.8, 0.7, 0.6, 0.55, 0.5 and 0.45 listed from left to right. The mode-coupling temperature 
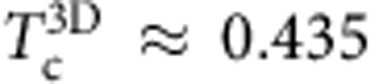
. The inset shows a trajectory plot of one small particle at *T*=0.45 where the colour of the trajectory plot changes when the particle moves more than one large particle diameter *σ* (length of the black line) over a time of 0.1*τ*_*α*_. (**b**) The mean square displacement 〈*δr*^2^(*t*)〉 for the 3D system showing the same temperatures as in **a**. (**c**) The bond-orientational correlation function *C*_Q_(t) for the 3D glass former showing the same temperatures as in **a**. (**d**) The self-intermediate scattering function *F*_s_(*k*;*t*) for the 2D glass former for *T*=1.0, 0.8, 0.7, 0.6, 0.5 and 0.45 listed from left to right. The inset shows a trajectory plot of a small particle, and no sudden jumps are observed. (**e**) The mean square displacement 〈*δr*^2^(*t*)〉 for the 2D system showing the same temperatures as in **d**. (**f**) The bond-orientational correlation function *C*_Ψ_(*t*) for the 2D glass former showing the same temperatures as in **d**. (**a**–**f**) Results for the Kob–Andersen binary mixtures.

In 3D, the dominant feature in the dynamics of deeply supercooled glass-forming fluids is transient localization of individual particles^6^, which is illustrated in the inset to Fig. 1a. The transient localization results in characteristic plateaus of the self-intermediate scattering function, 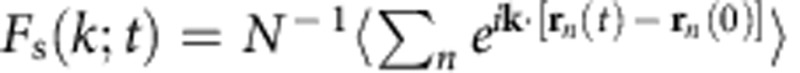 (|**k**|=*k*=7.2 in 3D and 6.28 in 2D), shown in Fig. 1a, and the mean square displacement, 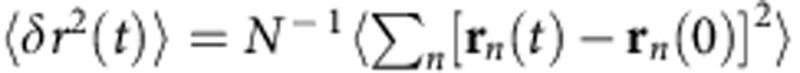, shown in Fig. 1b. The plateaus extend to longer and longer times on approaching the glass transition. Similar plateaus are observed in the collective scattering function, 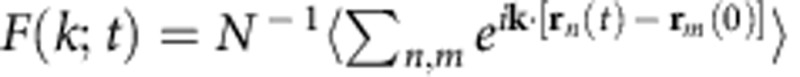, which describes relaxation of the density field (not shown), and in the correlation function quantifying bond-orientational correlations in 3D, *C*_Q_(*t*), shown in Fig. 1c (see Methods for the definition of *C*_Q_). Qualitatively similar slowing down of the translational and bond-orientational relaxation in 3D glass-forming fluids is analogous to the simultaneous appearance of translational and rotational long-range order in 3D crystalline solids.

The transient localization observed in 3D glassy dynamics is absent in 2D, as showed in the inset to Fig. 1d. Correspondingly, there is no intermediate time plateau in the self-intermediate scattering function in 2D (Fig. 1d). The final decay of *F*_s_(*k*; *t*), which in 3D is well described by a stretched exponential, is replaced by a very slow decay in 2D. The intermediate time plateau in the mean square displacement observed in 3D is replaced by an extended sub-diffusive regime in 2D (Fig. 1e). However, an intermediate time plateau is observed in the correlation function quantifying bond-orientational correlations in 2D, *C*_Ψ_(*t*) (Fig. 1f; see Methods for the definition of *C*_Ψ_). Shown in Fig. 3a–c are *F*_s_(*k*;*T*) and *C*_Ψ_(*t*) for three additional 2D glass formers and they behave similarly. Qualitatively different behaviour of the translational and bond-orientational relaxation in 2D glass-forming fluids is analogous to the absence of the translational and the presence of the bond-orientational long-range order in 2D solids^1^.

**Figure 2:**
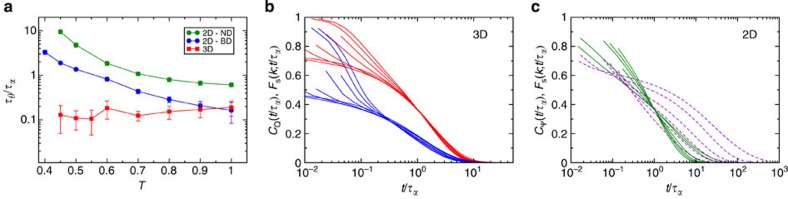
Bond angle and translational relaxation times. (**a**) The ratio of the relaxation time for the bond-orientational correlation functions *τ*_*θ*_ and the self-intermediate scattering function *τ*_*α*_ for the 2D system (circles) and the 3D system (squares). The ratio *τ*_*θ*_/*τ*_*α*_ for the 3D system is approximately constant and equal to 0.1–0.2 over the entire range of temperatures. The green circles are for Newtonian dynamics (ND) and the blue circles are results for Brownian dynamics (BD). The error bars are the standard error computed from four independent trajectories. (**b**) The self-intermediate scattering function *F*_s_(*k*; *t*) (red lines) and the bond angle time correlation function *C*_*Q*_(*t*) (blue lines) rescaled by *τ*_*α*_ for the 3D system. (**c**) The self-intermediate scattering function *F*_s_(*k*;*t*) (green solid lines) and the bond angle time correlation function *C*_Ψ_(*t*) (violet dashed lines) rescaled by *τ*_*α*_ for the 2D system. (**a**–**c**) Results for the Kob–Andersen binary mixtures.

**Figure 3:**
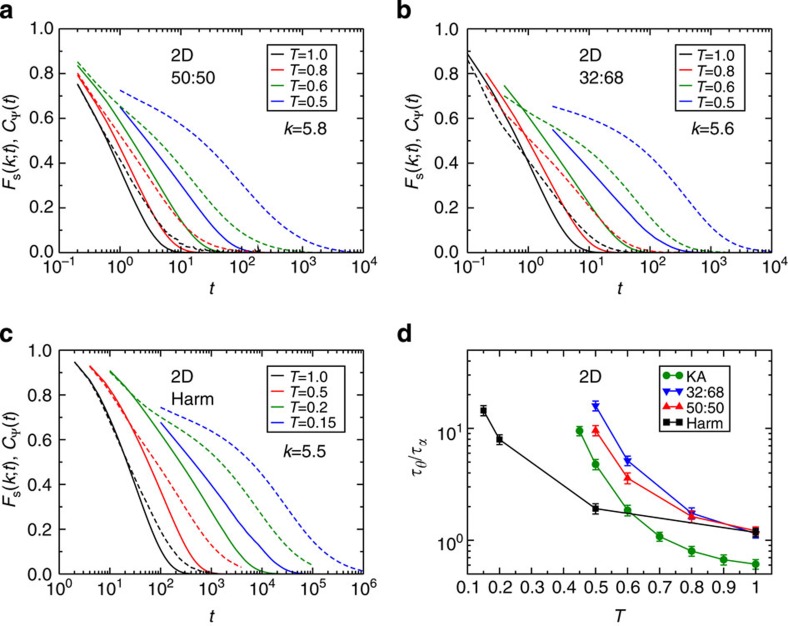
Translational and bond-orientational correlation functions in 2D. (**a**–**c**) The self-intermediate scattering function *F*_s_(*k*;*t*) (solid lines) and the bond-orientational correlation function *C*_Ψ_(*t*) (dashed lines) for three additional systems in 2D. The labels denote which system, and the systems are described in Methods. (**d**) The temperature dependence of the ratio of the bond angle relaxation time *τ*_*θ*_ to the structural relaxation time *τ*_*α*_.

To quantify decoupling between translational and bond-orientational relaxation, we compare the temperature dependence of the relaxation times characterizing *F*_s_(*k*;*t*) and *C*_(Q,Ψ)_(*t*), where *Q* and Ψ refer to 3D and 2D correlation functions, respectively. We define the translational relaxation time *τ*_*α*_ through the relation *F*_s_(*k*;*τ*_*α*_)=*e*^−1^ and the bond-orientational relaxation time *τ*_*θ*_ through *C*_(Q,Ψ)_(*τ*_*θ*_)=*e*^−1^. At the highest temperatures, the ratio *τ*_*θ*_/*τ*_*α*_ is less than one for both the 3D and the 2D glass former (Fig. 2a). However, this ratio stays approximately constant with decreasing temperature for the 3D glass former, but grows monotonically for the 2D glass former. In Fig. 3a–c, we show *F*_s_(*k*;*t*) and *C*_Ψ_(*t*) for three other 2D glass formers, which demonstrates that the decoupling of the temperature dependence of the translational and the bond-orientational relaxation time is general feature of 2D glassy dynamics. In addition, in Fig. 2b,c, we show that the final translational and orientational relaxation satisfies the time–temperature superposition in 3D but not in 2D, and we show corresponding figure for the 32:68 mixture (see Methods) in 3D in Supplementary Fig. 1 and for 2D in Supplementary Fig. 2. Figure 2c clearly demonstrates the decoupling of the temperature dependence of the translational and bond-orientational relaxation times in 2D.

### Dynamic heterogeneities

The non-exponential decay of *F*_s_(*k*;*t*) is frequently attributed to the emergence of domains, referred to as dynamic heterogeneities, in which the relaxation is spatially correlated and significantly different (faster or slower) than the average relaxation. While we find non-exponential decay in *F*_s_(*k*;*t*) for 3D and 2D glass formers, the nature of the decay is very different and this difference is mirrored by differences in the heterogeneous dynamics.

**Figure 4:**
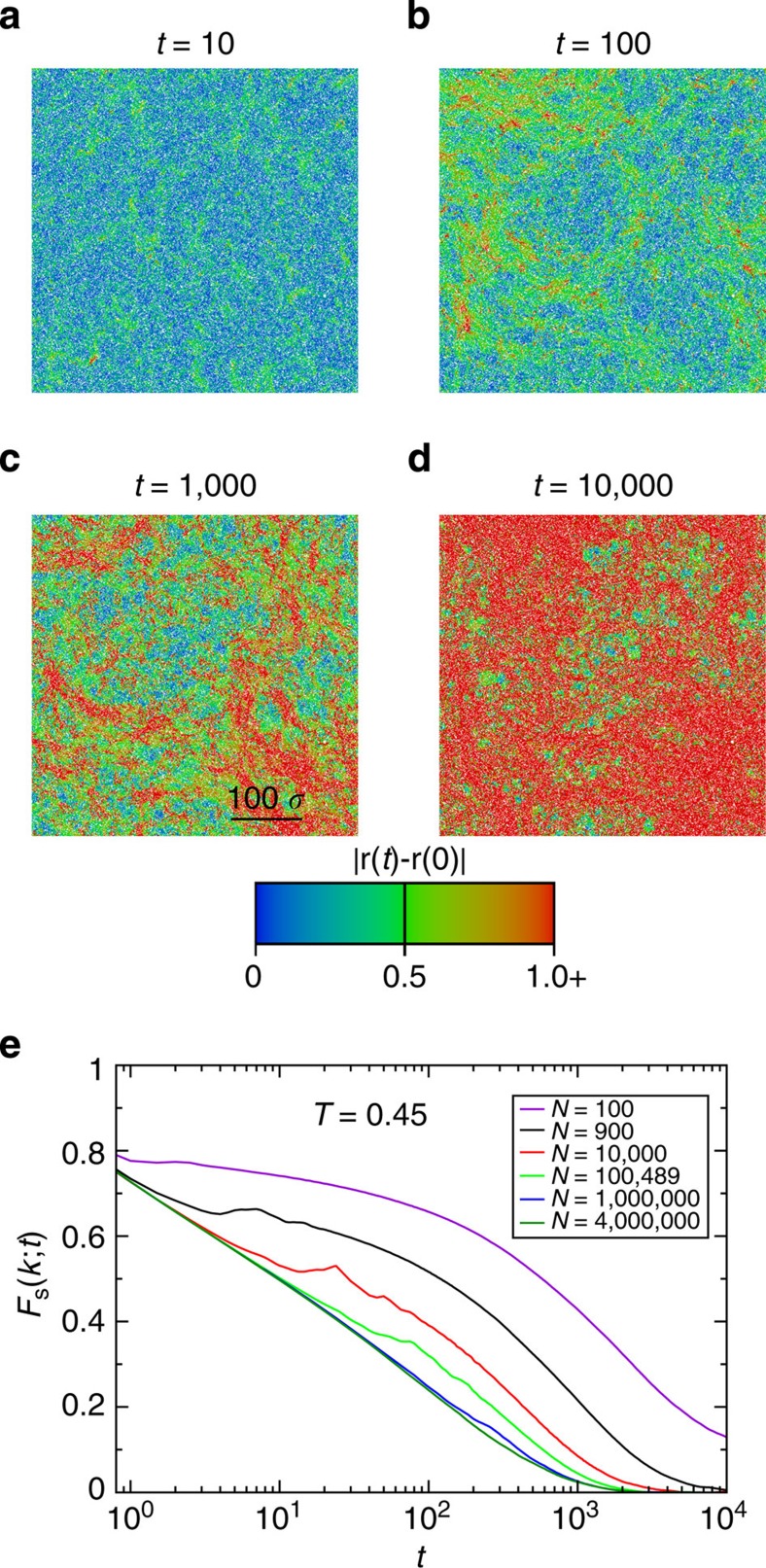
Dynamic heterogeneities. (**a**–**d**) Displacement maps of the centre of a system of 4 million particles that show the position of the particles at *t*=0 coloured by the displacement of the particle at a later time *t* for *T*=0.45. There are ∼250,000 particles in each map. The scale bar in **d** corresponds to 100 larger particle diameters. (**e**) The self-intermediate scattering function *F*_s_(*k*; *t*) calculated for systems of 100 particles to 4 million particles. There are clear finite size effects for less than 1 million particles. (**a**–**e**) Results are for the 2D Kob–Andersen mixture.

Shown in Fig. 4a–d are displacement maps showing the centre of a four million particle simulation in 2D at *T*=0.45. The maps are created by colouring the particles, whose position is shown at *t*=0, according to the magnitude of their displacements |**r**_*n*_(*t*)−**r**_*n*_(0)| between times 0 and *t*. The red particles have moved a distance equal to or greater than the diameter of a larger particle. There are large domains of particles that have moved less than a particle diameter even at *t*=10,000.

Considering the large dynamically heterogeneous regions in Fig. 4a–d, it is unsurprising that we also find large finite size effects. Shown in Fig. 4e is *F*_s_(*k*;*t*) calculated for different size systems at the same temperature as shown in Fig. 4a–d. A plateau reminiscent of the plateau in 3D systems is present for the smaller systems but gradually disappears with increasing system size. Similar finite size effects are also evident in the mean square displacement (Supplementary Fig. 3) and the inherent structure dynamics (Supplementary Fig. 4).

To quantify dynamic heterogeneity shown in Fig. 4a–d, we use a four-point structure factor *S*_4_(*q*;*t*)^15^ constructed from overlap functions *w*_*n*_(*a*;*t*)=Θ(*a*−|**r**_*n*_(*t*)−**r**_*n*_(0)|), where Θ(·) is Heaviside's step function. The parameter *a* is chosen such that 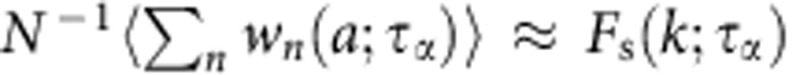, which results in *a*=0.25 in 3D and *a*=0.22 in 2D. To characterize the slow domains, we calculate 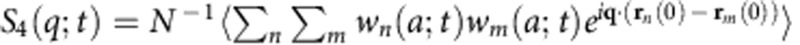 (note that *w*_*n*_(*a*;*t*) restricts the sums over the particles that moved less than *a* over a time *t*). The characteristic size of dynamically heterogeneous regions is quantified through the dynamic correlation length *ξ*_4_(*t*), which is determined from fitting *S*_4_(*q*; *t*) for small *q* to the Ornstein–Zernicke form *χ*_4_(*t*)/{1+[*q*ξ_4_(*t*)]^2^}. Here *χ*_4_(*t*) is the dynamic susceptibility, which characterizes the overall strength of the dynamic heterogeneity.

**Figure 5:**
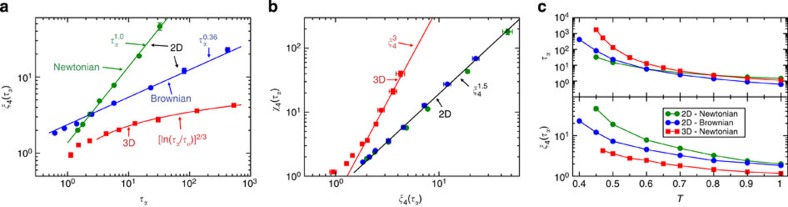
Properties of dynamic heterogeneities. (**a**) The dynamic correlation length *ξ*_4_(*τ*_*α*_) versus the translational relaxation time *τ*_*α*_. The circles are the results for the 2D system where the underlying dynamics is Newtonian (green) and Brownian (blue). The lines are fits to a power law 
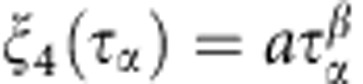
. The red squares are results for the 3D system where the underlying dynamics is Newtonian. The red line is a fit to *ξ*_4_(*τ*_*α*_)=*b*[ln(*τ*_*α*_/*τ*_*o*_)]^2/3^. (**b**) The dynamic susceptibility *χ*_4_(*τ*_*α*_) versus the dynamic correlation length *ξ*_4_(*τ*_*α*_) for the 2D system where the underlying dynamics are Newtonian (green circles) and Brownian (blue circles). The red squares are results for the 3D system. The lines are fits to the power laws *χ*_4_(*τ*_*α*_)∼*ξ*_4_(*τ*_*α*_)^3^ in 3D and *χ*_4_(*τ*_*α*_)∼*ξ*_4_(*τ*_*α*_)^1.5^ in 2D. (**c**) The relaxation time *τ*_*α*_ and the dynamic correlation length *ξ*_4_(*τ*_*α*_) where the underlying dynamics are Newtonian (green) and Brownian (blue) for the 2D system, and Newtonian for the 3D system (red). The error bars in all the figures are standard errors computed from four independent trajectories. (**a**–**c**) Results are for the Kob–Andersen mixtures.

In Fig. 5a, we show the correlation between the translational relaxation time, *τ*_*α*_, and the dynamic correlation length calculated at *τ*_*α*_, *ξ*_4_(*τ*_*α*_), for the 3D and 2D glass-forming fluids. While for the 3D system we find that a power law is a poor description for an extended range of *τ*_*α*_, and a better description is *ξ*_4_(*τ*_*α*_)∼[In(*τ*_*α*_/*τ*_0_)]^2/3^ (red line in Fig. 5b), we find that a power law 
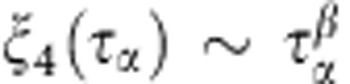
 with *β*=1.0±0.1 describes the full range of results well for the 2D system. We show results for an additional glass former in 2D and 3D, the 32:68 mixture, in Supplementary Fig. 5. Note that similar power-law behaviour was observed in simulations of 2D granular fluids^10^. In Fig. 5b, we show that the relationship between the dynamic susceptibility and the dynamic correlation length is fundamentally different in 3D and 2D. For 3D systems, *χ*_4_(*τ*_*α*_)∼*ξ*_4_(*τ*_*α*_)^3^ at low temperatures, which implies compact dynamically heterogeneous regions. For 2D systems, we observe *χ*_4_(*τ*_*α*_)∼*ξ*_4_(*τ*_*α*_)^1.5^, which suggests more ramified dynamically heterogeneous regions, see Fig. 4d.

### Dependence on the microscopic dynamics

**Figure 6:**
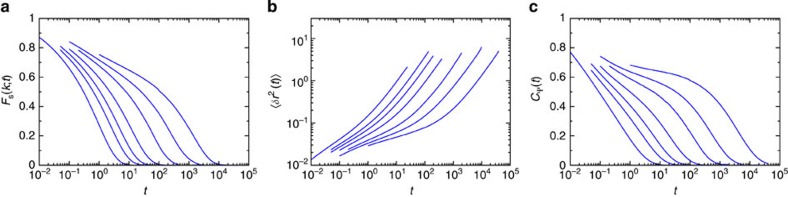
Structural relaxation in two dimensions with Brownian dynamics. (**a**) The self-intermediate scattering function *F*_s_(*k*; *t*) for *T*=1.0, 0.8, 0.7, 0.6, 0.5, 0.45 and 0.4 listed from left to right. (**b**) The mean square displacement 〈*δr*^2^(*t*)〉 showing the same temperatures as in **a**. (**c**) The bond angle time correlation function *C*_Ψ_(*t*) showing the same temperatures as in **a**. We emphasize that the six higher temperatures showed in this figure are the same as the temperatures showed in Fig. 1d–f. The finite size effects are much more pronounced in systems evolving with Newtonian dynamics. This fact made impossible to simulate the 2D system at *T*=0.4 with Newtonian dynamics. (**a**–**c**) Results are for the 2D Kob–Andersen mixture.

Last, we discuss the dependence of the long-time relaxation in 2D on the underlying microscopic dynamics and two important consequences. In 3D, an important finding is that the long-time dynamics does not depend on the microscopic dynamics; the same long-time dynamics has been observed in simulations using Newtonian^7^, stochastic^11^, Brownian^12^ and Monte Carlo^13^ dynamics. This result can be rationalized within the mode-coupling approach^14^. Surprisingly, we find that in 2D the long-time dynamics is quite different in the case of microscopic Newtonian and Brownian dynamics. The results corresponding to the those shown in Fig. 1d–f for the Newtonian case are shown in Fig. 6 for the Brownian case. Notably, the decay of *F*_s_(*k*;*t*) is strikingly different for the Brownian simulations than for the Newtonian simulations.

Importantly, the temperature dependence of the translational relaxation time is also decoupled from orientational relaxation time in the case of Brownian dynamics (Fig. 2a) but the ratio *τ*_*θ*_/*τ*_*α*_ is not as large for Brownian dynamics than for Newtonian dynamics. In addition, we find a power-law relationship 
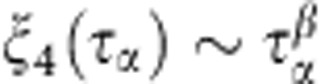
 between the dynamic correlation length and the relaxation time but with *β*=0.36±0.05, which is a different exponent than obtained for Newtonian dynamics, see Fig. 5a. However, we find that the relationship between the strength of the dynamic heterogeneity and the dynamic correlation length in 2D is the same for Brownian and Newtonian dynamics, see Fig. 5b. The latter two results show that the universality of the relationships between the relaxation time and properties of heterogeneous dynamics that we found in 3D^15^ is absent in 2D. Furthermore, a full description of heterogeneous dynamics in 2D must also include the influence of the microscopic dynamics, and descriptions solely in terms of the structure or the potential energy landscape are not sufficient in 2D.

## Discussion

Glassy dynamics in 2D and in 3D are profoundly different. We presented detailed results for one glass former and we verified that the features of the translational relaxation and dynamic heterogeneity are qualitatively the same for three additional 2D glass formers. Our results call for a re-examination of the present glass transition paradigm in 2D. We note that there is currently no theoretical framework that accounts for the different dynamics observed in the 2D glass-forming systems. However, we note that the dynamic picture of the random first-order transition theory breaks down for dimensions less than two, and has been described as marginal for two dimensions^16^,^17^. Moreover, insights gained from theoretical analysis of the 2D glassy dynamics and glass transition might shed light onto slow dynamics and the glass transition in 3D. It will also be interesting to investigate if the differences between 2D and 3D glassy dynamics are observable for glass-forming fluids in confinement and at interfaces or surfaces, that is, for quasi 2D systems.

## Methods

### Simulations

We simulated binary mixtures of Lennard–Jones particle in two and three dimensions. The interaction potential is *V*_*αβ*_(*r*)=4ɛ_*αβ*_[(*σ*_*αβ*_/*r*)^12^−(*σ*_*αβ*_/*r*)^6^] where ɛ_*BB*_=0.5ɛ_*AA*_, *ɛ*_*AB*_=1.5ɛ_*AA*_, *σ*_*BB*_=0.88*σ*_*AA*_ and *σ*_*AB*_=0.88*σ*_*AA*_. The results are presented in reduced units where *σ*_*AA*_≡*σ* is the unit of length and ɛ_*AA*_ the unit of energy. The unit of time for the Newtonian dynamics simulations is 
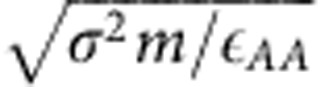
 and the mass *m* is the same for both species. The Newtonian dynamics simulations^9^ were performed using LAMMPS^18^ for the 2D and 3D simulations and HOOMD blue for the 2D simulations^19^. The LAMMPS simulations were run in an NVE ensemble but there is significant energy drift for the HOOMD blue simulations for the lowest temperatures. Therefore, we ran the HOOMD blue simulations using an NVT Nosé–Hoover thermostat with a coupling constant *τ*=10. We ran at least one LAMMPS NVE simulation at every temperature to make sure that the conclusions did not depend on the thermostat. All the results are averages over four or more production runs. The equations of motion for the Brownian dynamics simulations^9^ are 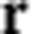
_*n*_(*t*)=*γ*^−1^**F**_*n*_(*t*)+***η***_*n*_(*t*), where *γ*=1 is the friction coefficient, **F**_*n*_(*t*) is the force on particle *n* at time *t* and ***η***_*n*_ is a random noise term. The random noise satisfies the fluctuation dissipation relation 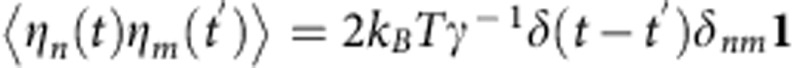, where **1** is the unit tensor. The unit of time for the Brownian dynamics simulation is *σ*^2^*γ*/ɛ_*AA*_. The Brownian dynamics simulations were run using a modified version of LAMMPS and our in house developed code. Inherent structure trajectories were created by quenching the system to the local potential energy minimum using the FIRE algorithm^20^.

We simulated 2D systems of 10,000 particles for *T*⩾0.9 and 250,000 particles for 0.5≤*T*≤0.8. At *T*=0.45, we studied 4 million particles for the Newtonian dynamics simulations, but 250,000 particles for the Brownian dynamics simulations. We simulated 27,000 particles in 3D using Newtonian dynamics. To check that the results are independent of the system size for each state point for the 2D Newtonian dynamic simulations, we ran 100,489 particle simulations and checked to see if the results agreed with the 250,000 particle simulations. At *T*=0.45 they did not agree, and we increased the system size until we found agreement between the 4 million particle system and an 8 million particle system. For the 2D Brownian dynamics simulations, we found agreement between 10,000 particle simulations and 250,000 particle simulations for *T*⩾0.45. For *T*=0.4, we found that a 100,489 particle simulation agreed with a 250,000 particle system.

We also examined the translational dynamics, bond-orientational and dynamic heterogeneities for three additional systems in 2D and one additional glass-forming system in 3D. The first system is the one studied in ref. 21 and consists of a 31.67:68.33 binary mixture with the potential *V*_*αβ*_(*r*)=ɛ(*σ*_*αβ*_/*r*)^12^. The size ratios are *σ*_*AB*_=1.1*σ*_*AA*_ and *σ*_*BB*_=1.4*σ*_*AA*_. We simulated this system using 250,000 particles in 2D and 100,000 particles 3D. The number density 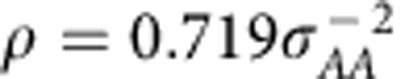 in 2D and 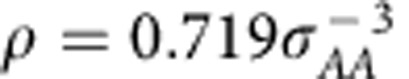 in 3D. The units for energy is ɛ, length is *σ*_*AA*_, time is 
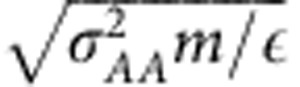
, and temperature is ɛ/*k*_*B*_. We denote this system with the label 32:68.

The second system was introduced in ref. 22, and consists of an 50:50 mixture of repulsive particles where the potential *V*_*αβ*_(*r*)=ɛ(*σ*_*αβ*_/*r*)^12^. The size ratios are given by *σ*_*AB*_=1.2*σ*_*AA*_ and *σ*_*BB*_=1.4*σ*_*AA*_ and the number density 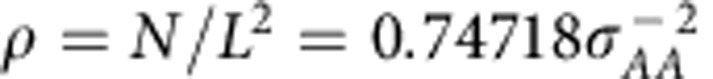. We simulated 250,000 particles for this second additional system. The units for energy is ɛ, length is *σ*_*AA*_, time is 
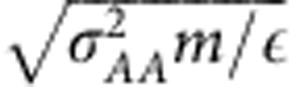
 and temperature is ɛ/*k*_*B*_. We denote this system with the label 50:50.

The third system is the one introduced in ref. 23, which consists of an 50:50 mixture of harmonic spheres with the interaction potential *V*_*αβ*_(*r*)=0.5ɛ(1−*r*/*σ*_*αβ*_)^2^ for 
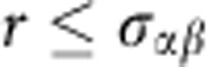
 and *V*_*αβ*_(*r*)=0 otherwise. The size ratios are given by *σ*_*AB*_=1.2*σ*_*AA*_ and *σ*_*BB*_=1.4*σ*_*AA*_ and 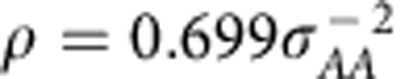. We simulated 250,000 particles for this third additional system. The units for energy is ɛ, length is *σ*_*AA*_ and time is 
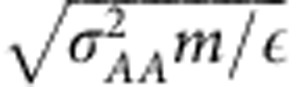
. The unit for temperature is 10^−3^ɛ/*k*_*B*_. We denote this system with the label Harm.

### Bond-orientational correlation functions

To measure bond-orientational relaxation times in 2D, we first define 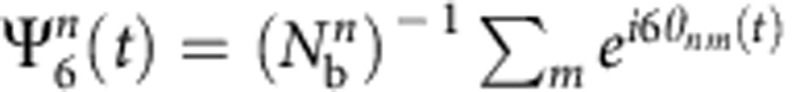, where *θ*_*nm*_(*t*) is the angle between particle *n* and particle *m* at a time *t*, 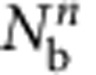 is the number of neighbours of particle *n*, and the sum is over the neighbours of particle *n* at the time *t*. The neighbours are determined through Voronoi tessellation^3^. The time dependence of the bond angle correlations was monitored by calculating 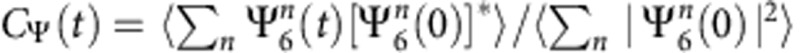, where * denotes the complex conjugate.

To measure bond-orientational relaxation in 3D, we define 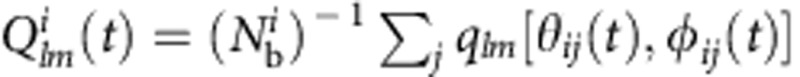, where *q*_*lm*_(*θ*,*φ*) are the spherical harmonics^24^ and the sum is over the neighbours of a particle *i* at a time *t* determined through Voronoi tessellation. Next, we define the correlation function 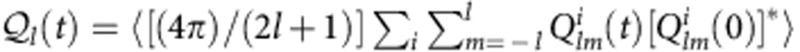. We calculated *C*_Q_(*t*)=_6_(*t*)/_6_(0) to monitor the decay of orientational correlations.

We note that the conclusions remain unchanged if we define neighbours as being less than a distance equal to the first minimum of the pair correlation function rather than through Voronoi tessellation.

## Additional information

**How to cite this article:** Flenner, E. & Szamel, G. Fundamental differences between glassy dynamics in two and three dimensions. *Nat. Commun.* 6:7392 doi: 10.1038/ncomms8392 (2015).

## Supplementary Material

Supplementary Figures Supplementary Figures 1-5
