# Supplementary material for: Fundamental differences between glassy dynamics in two and three dimensions
Source: Nat Commun. 2015 Jun 12;6:7392. doi: 10.1038/ncomms8392 (PMC4490572; doi:10.1038/ncomms8392)
Supplement: Supplementary Figures — 1-5 [file ncomms8392-s1.pdf]

Supplementary Figures

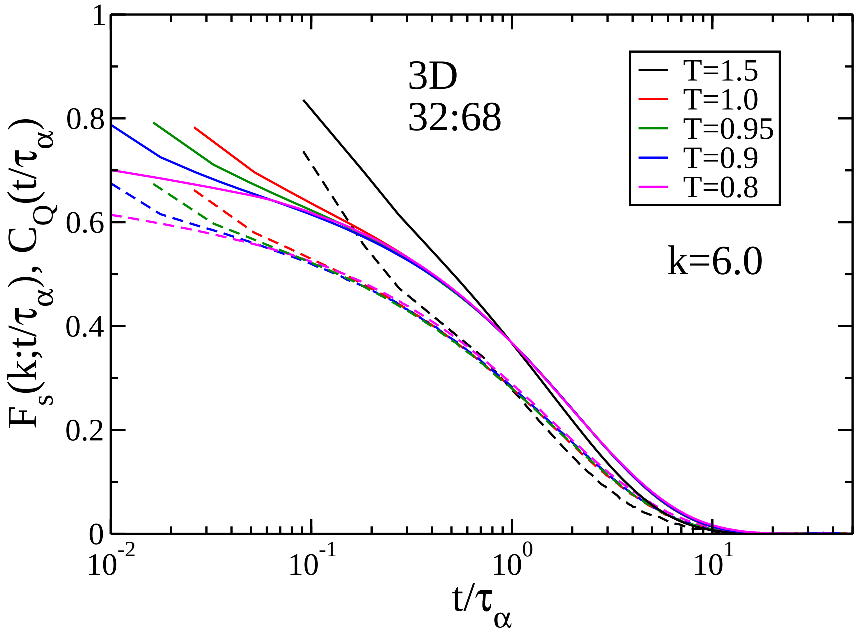

Supplementary Figure 1: Translational and bond-orientational relaxation in 3D. The self-intermediate scattering function $F_{\rm s}(k;t)$ (solid lines) and the bond-orientational correlation function $C_{\rm Q}(t)$ (dashed lines) rescaled by the alpha-relaxation time τ_α in 3D. We define τ_α as when $F_{\rm s}(\tau_\alpha) = e^{-1}$. The system is the 32:68 system described in Methods.

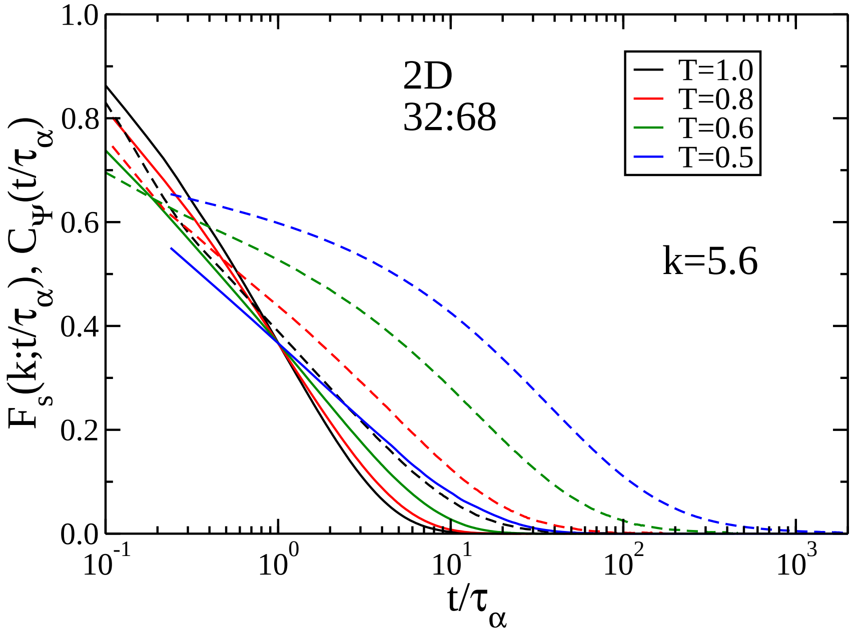

Supplementary Figure 2: Translational and bond-orientational relaxation in 2D. The self-intermediate scattering function $F_{\mathrm{s}}(k;t)$ (solid lines) and the bond-orientational correlation function $C_\Psi(t)$ (dashed lines) rescaled by the alpha-relaxation time τ_α in 2D. We define τ_α as when $F_{\mathrm{s}}(\tau_\alpha) = e^{-1}$. The system is the 32:68 system described in Methods.

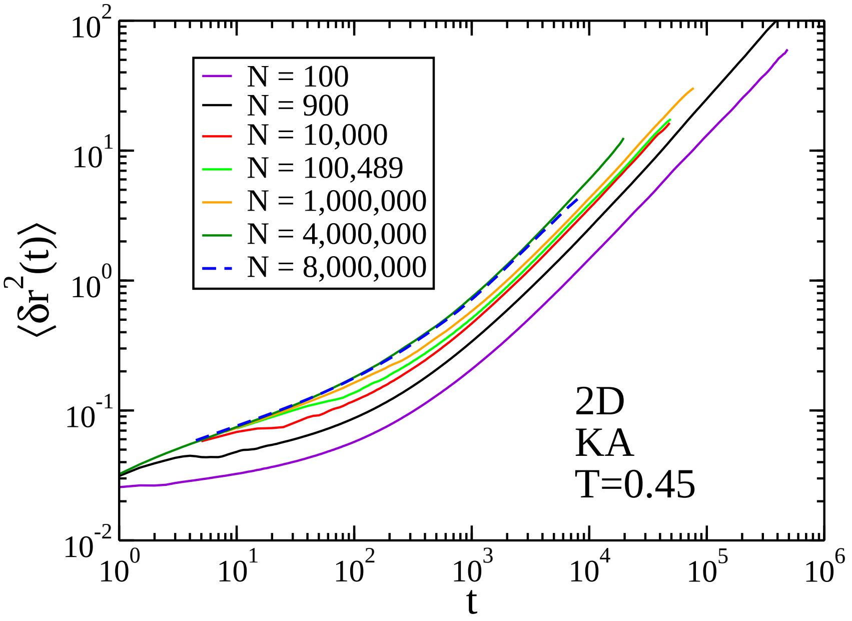

Supplementary Figure 3: Mean square displacement finite size effects. The mean square displacement for the two-dimensional KA system for different system sizes at $T = 0.45$.

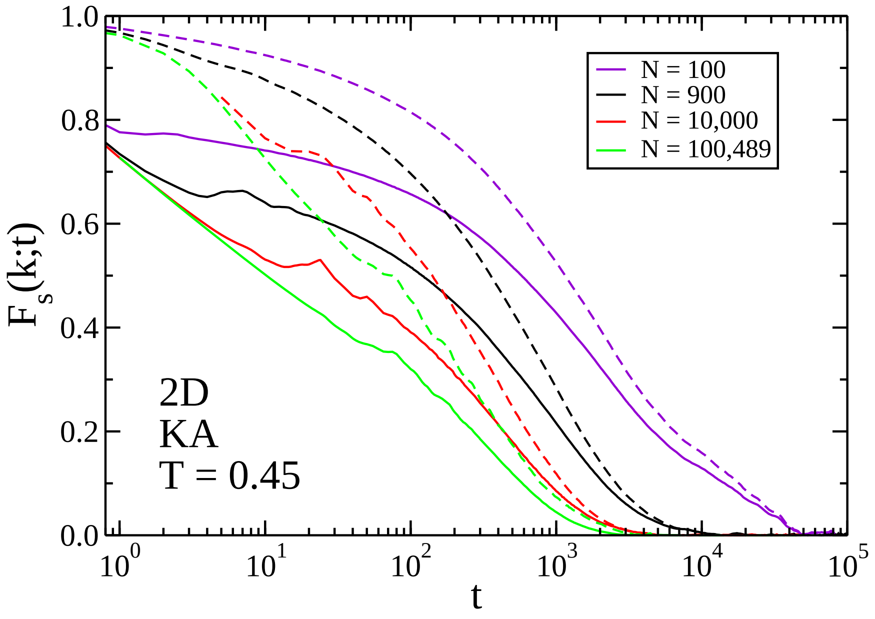

Supplementary Figure 4: Inherent structure finite size effects. The self-intermediate scattering function for the standard Newtonian dynamics (solid lines) and the inherent structure dynamics (dashed lines) for the two-dimensional KA system for different system sizes at $T = 0.45$.

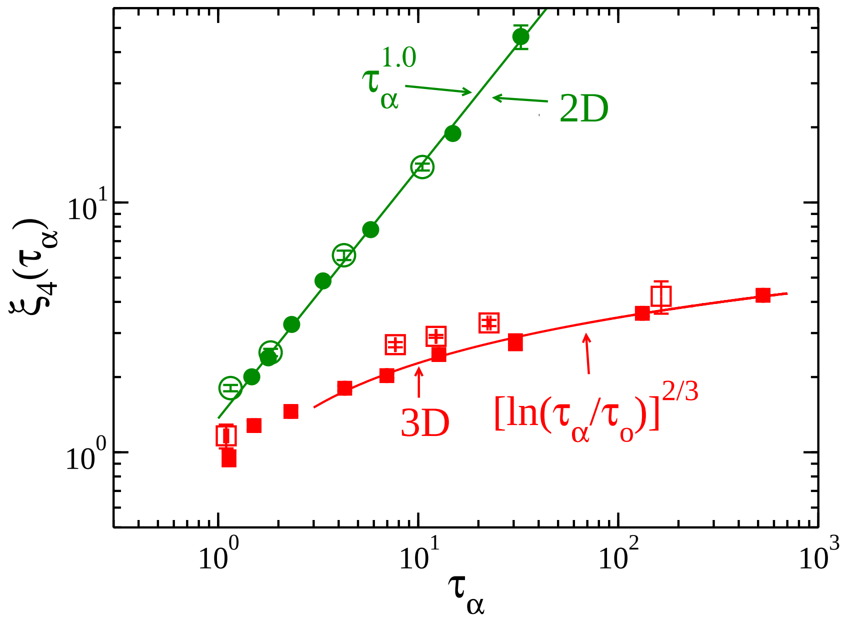

Supplementary Figure 5: Dynamic correlation length in 2D and 3D. The dynamic correlation length $\xi_4(\tau_\alpha)$ versus the alpha-relaxation time τ_α for the KA system (filled symbols) and the 32:68 system (open symbols) in 2D (green circles) and 3D (red squares).
